# Phytochemicals in the Control of Human Appetite and Body Weight

**DOI:** 10.3390/ph3030748

**Published:** 2010-03-22

**Authors:** Sonia A. Tucci

**Affiliations:** Kissileff Laboratory for the Study of Human Ingestive Behaviour, School of Psychology, University of Liverpool, Eleanor Rathbone Building, Bedford Street South, Liverpool L69 7ZA, UK; Email: sonia.tucci@liv.ac.uk; Tel.: +44-151-794-1121; Fax: +44-151-794-2945

**Keywords:** obesity, calories, energy intake, energy expenditure

## Abstract

Since obesity has grown to epidemic proportions, its effective management is a very important clinical issue. Despite the great amount of scientific effort that has been put into understanding the mechanisms that lead to overconsumption and overweight, at the moment very few approaches to weight management are effective in the long term. On the other hand, modern society is also affected by the growing incidence of eating disorders on the other side of the spectrum such as anorexia and bulimia nervosa which are equally difficult to treat. This review will try to summarise the main findings available in the literature regarding the effect of plants or plant extracts (phytochemicals) on human appetite and body weight. The majority of plant extracts are not single compounds but rather a mixture of different molecules, therefore their mechanism of action usually targets several systems. In addition, since some cellular receptors tend to be widely distributed, sometimes a single molecule can have a widespread effect. This review will attempt to describe the main phytochemicals that have been suggested to affect the homeostatic mechanisms that influence intake and body weight. Clinical data will be summarised and scientific evidence will be reviewed.

## 1. Phytochemicals and Weight Control

Body weight maintenance can be achieved through manipulation of energy expenditure (EE, mainly heat production also known as thermogenesis), appetite suppression/satiety enhancement, and fat and glucose absorption blocking. Either one or more frequently, several components can be altered by the phytochemicals described below. 

Phytochemicals are found in food items and herbal preparations where they could alter appetite beyond the effects expected by normal nutrient loads. This added to the fact that they can exert far fewer side effects, may provide an alternative treatment or could be used to enhance the effect of prescription medications. This review will focus on human studies on the effects of phytochemicals in the regulation of appetite and body weight. Although most phytochemicals that affect body weight regulation have a complex mechanism of action, for the purpose of this review they will be grouped according to their main effect (increase or decrease body weight) and the site of main mechanism of action (central nervous system (CNS), peripheral or both). 

## 2. Phytochemicals that Decrease Body Weight Mainly through a Peripheral Mechanism

### 2.1. Korean Pine Nut Oil

Korean pine nut oil (Pinnothin^®^) is obtained by natural pressing of Korean pine nuts (*P. koraiensis*) and it contains triglycerides (TG) and more than 92% poly- and mono-unsaturated fatty acids (PUFAs and MUFAs) like pinolenic acid (C18:3), linoleic acid (C18:2) and oleic acid (C18:1) [1]. Korean pine nut oil is claimed to be unique in that it contains approximately 15% pinolenic acid (C18:3). Korean pine nut free fatty acids (FFA) significantly increase the release of satiety hormones such as cholecystokinin (CCK) [2]. CCK delays gastric emptying and produces a subsequent increased feeling of satiety and a decreased appetite. In terms of inducing satiety-hormone secretion, long chain fatty acids are more effective than medium chain fatty acids, PUFAs are more effective than MUFAs [3,4]. 

Pine nut FFA administration to overweight postmenopausal women has reported to produce a significant increase of CCK-8 and glucagon like peptide-1 (GLP-1). The appetite sensation "prospective food intake" and “desire to eat” are also lowered, and these effects last up to 4 hours [2,5]. Administration of Korean pine nut FFA (2 grams) reduced lunch *ad-libitum* food intake by 9% compared with the placebo control. This was achieved without increasing caloric intake during the evening meal which suggests there is no compensation for the lesser food intake during lunch [6]. So far no adverse effect of the compound during the study period or at post study debriefing have been reported. 

### 2.2. Palm Oil + Oat Oil Fractions

Olibra^®^ is a fat emulsion formulated from palm oil (40%) and oat oil fractions (2.5%). Its mechanism of action is similar to that of Korean pine nut oil, increasing and prolonging the release of peptide YY, CCK and GLP-1 [7,8] which inhibit upper gut motility (to slow gastric emptying and intestinal transit) generating an indirect satiety effect [9,10].

Compared to other appetite suppressant products, the evidence supporting the effects of Olibra^®^ is more comprehensive. Double-blind, placebo-controlled reports indicate that Olibra^®^ administration to lean, overweight and obese individuals significantly reduced hunger and desire to eat [11] with a consequent decrease of energy and macronutrient intake up to 36 h post-consumption [7,12]. The results obtained with chronic administration suggest that Olibra^®^ could help to maintain weight after weight loss programmes [8]. Taken together these findings indicate that in addition to having acute effects on energy intake (EI) and hunger/satiety, Olibra^®^ could be beneficial for weight maintenance.

### 2.3. Garcinia Cambogia

*G. cambogia* is a tree indigenous to southeast Asia. Commercially available (−)- hydroxycitric acid (HCA) extract from *G. cambogia* [Super CitriMax^®^ HCA-SXS (HCA-SX^®^)] is mainly extracted from the dried and cured pericarp of the fruit of this species [13]. These pericarp rinds have been used for centuries in regional cooking practices and are reported to make meals more filling and satisfying [14,15], without any reported harmful effects. HCA may promote weight reduction through suppressed de novo fatty acid synthesis, increased lipid oxidation and reduced food intake [16]. Enhanced satiety may also account for the reported suppression of energy consumption but this has yet to be demonstrated. 

Studies investigating the effects of HCA in humans have provided conflicting results. While some reports have shown that administration of HCA (1.2–1.5 g/day) to overweight participants did not produce any significant decrease in body weight or appetite variables [17,18], others reported that daily administration of a relatively low dose of HCA (900 mg/day) over two weeks, reduced EI and sustained satiety. It is important to note that the reduction in body weight reported in this study was only minor [19]. Preuss *et al*. [20] found that eight week administration of 2,800 mg/day of HCA produced a reduction of 5.4% and 5.2% in baseline body weight and BMI respectively compared to controls. HCA at those doses also reduced food intake, total cholesterol, LDL, TG and serum leptin levels, and increased HDL levels and excretion of urinary fat metabolites (a biomarker of fat oxidation). The available literature seems to support the claim that HCA may be more effective at moderating weight gain [21] than at promoting weight loss, making the compound potentially more useful for weight maintenance after an initial loss. It is important to note that some clinical studies with HCA have encountered mild adverse events such as headache, and upper respiratory tract and GI symptoms [18]. 

## 3. Phytochemicals that Block Pancreatic Lipase and α-Amylase

Dietary fat is the most energy dense macronutrient, and most closely linked to overweight and obesity. Therefore, the blockage of fat absorption is a logical target for obesity treatment. Currently, one of the most effective treatments is malabsorptive surgery [22], therefore it is not surprising that non-surgical approaches have focused on drugs that inhibit the absorption of macronutrients. The phytochemicals described below act by blocking the breakdown and consequent absorption of dietary carbohydrates and/or lipids.

### 3.1. Tea Cathechins

Three kinds of tea: oolong, green, and black, are widely used as traditional healthy drinks all over the world and green and oolong tea have been traditionally reported to have anti-obesity and hypolipidemic actions. Black tea also contains many active ingredients [23]; however some of these may not survive processing. 

#### 3.1.1. Oolong Tea

Catechins in oolong tea are reported to prevent obesity by two main mechanisms: the inhibition of small-intestine micelle formation and the inhibition of α-glucosidase activity which would lead to a decrease in carbohydrate absorption [24]. In a double-blind, placebo-controlled study, twelve weeks daily administration of oolong tea (containing 690 mg of catechins) to normal and overweight males (with daily EI set at 90% of recommended caloric intake) produced a significant reduction in body weight (1.5%), body mass index (BMI) (1.5%), waist circumference (2.0%), and body fat mass (3.7%), compared to the placebo group [25]. These results suggest that oolong tea consumption might be useful as an adjuvant during weight loss programmes.

#### 3.1.2. Green Tea

The long term consumption of green tea and its extract (GTE, commercially available as pills, patches, gums, mints, extracts, and ice creams) have been associated with weight loss mainly through a thermogenic mechanism [26]. The main active ingredients in GTE – the catechins epigallocatechin gallate (EGCG; Teavigo^®^), epigallocatechin (EGC), epicatechin gallate (ECG), and epicatechin (EC) – are responsible for many of the beneficial effects of green tea [27,28]. 

Administration of GTE to humans has been reported to decrease body weight (0.6 to –1.25 kg) and body fat 0.5 to –1.8 kg [29,30,31,32,33,34]. However, it is important to highlight that in some of these studies participants were also subjected to moderate energy restriction (90% of individual energy requirements) [25,35] or exercise [34]. Another study found that administration of GTE to overweight participants produced a 4.6% decrease in body weight compared to baseline [36]. Monitoring of food intake showed that GTE did not diminish EI between groups. Nevertheless the majority of these studies were uncontrolled, not blind and not strictly controlled for EI and physical activity. The lack of effect on EI could point to thermogenesis and fat oxidation as the main mechanisms responsible for weight loss [37,38,39]. There are reports that have shown that catechins from GTE have been associated with an increase in sympathetic nervous system activity, thermogenesis and fat oxidation in humans [25,37]. Certainly the effects of GTE on weight control are worthy of further clinical investigation.

### 3.2. Green Coffee Bean

Green coffee bean extract (GCBE) (Quest Green Coffee ^®^, Svetol^®^) contains 10% caffeine and 27% chlorogenic acid as the principal constituents. However, the roasting process of coffee drastically reduces the level of chlorogenic acid and its related compounds [40]. The administration of instant coffee enriched with chlorogenic acid to humans induced a reduction in body fat and body mass at least in part due to a reduction in the absorption of glucose [41]. The reduction of glucose absorption would ultimately lead to an increase in the consumption of fat reserves, due to the reduced availability of glucose as an energy source [41]. Since coffee drinking and obesity appear co-existing in most developed societies the efficacy of these products in those already regularly exposed to caffeine remains to be demonstrated. However, it is important to note that a major consequence of blocking digestion of carbohydrates in the proximal gut is colonic fermentation which leads to increased microbial production of gas in the bowel; this effect can limit its use.

### 3.3. Citrus Aurantium

*C. aurantium* (Citrus Aurantium extract ^®^, Bitter Orange extract^®^) contains alkaloids such as *p*‑octopamine and synephrines which exert adrenergic agonist activity [42] and are present in supplements designed to aid weight loss [43]. Synephrines could potentially increase EE and decrease food intake [44]. In addition, there is some evidence that adrenergic agonists, including *C. aurantium* synephrines, decrease gastric motility [45]. 

There are a few trials that have examined the effects of *C. aurantium* synephrines alone, or in combination with other ingredients, on body weight and/or body composition in humans. Overall, these studies reported a loss of 2.4–3.4 kg among participants using synephrines, while placebo groups lost 0.94–2.05 kg [46]. These results point towards some beneficial effects from synephrines supplementation, however they cannot be considered conclusive at this point because they do not separate the effects of *C. aurantium* or synephrines from those of other ingredients, particularly ephedrine. In addition these trials were of short duration and sample sizes were frequently inadequate. Due to the above mentioned drawbacks, it would be difficult to formulate *C. aurantium* related public health recommendations with confidence.

## 4. Phytochemicals that Decrease Body Weight through a Combination of Central and Peripheral Mechanisms

### 4.1. Caffeine

Caffeine (Caffeine Pro^®^) is the most widely consumed behaviourally active substance in the world [47]. Almost all caffeine consumed comes from dietary sources (beverages and food), most of it from coffee and tea [47]. The central effects of caffeine at habitually consumed doses are due to its effects on the widely distributed adenosine A1, A2A and A2B receptors [47,48]. 

Long-term intervention studies in humans have failed to report conclusive effects of caffeine consumption on body weight [49,50,51,52]. A possible explanation for the lack of a long-term effects could be the development of a tolerance to its effects [49]. Nevertheless, controlled experiments investigating its acute effect have found that caffeine has a small reducing effect on caloric intake [53,54,55,56]. Reintroduction of caffeine in regular coffee consumers after a period of abstinence was found to reduce the number of meals without affecting meal size, this lead to a decrease of EI [57]. Increased caffeine intake has been reported to produce slightly smaller weight gains in men and women when compared to controls [58]. 

Caffeine also seems to exert thermogenic and lipolytic actions [59]. However, both effects are reported to be more prominent in normal weight individuals [60,61,62]. To conclude, caffeine seems to act through central and peripheral mechanisms which would, over the long term, help with achieving weight loss. However, because of the issue of tolerance, the potential benefits of such an approach to weight control in societies of individuals already exposed to high levels of caffeine may be somewhat limited.

### 4.2. Nicotine

Nicotine is an alkaloid naturally occurring in tobacco leaves [63] and is their major addictive component. Similarly to caffeine, nicotine exerts its effect through central and metabolic actions. Among several effects, nicotine reduces appetite and alters feeding patterns typically resulting in reduced body weight [64]. Nicotine increases metabolic rate and decreasing metabolic efficiency. Smoking a single cigarette has been shown to induce a 3% rise in EE within 30 minutes [65]. In the CNS, nicotine modulates the central pathways that regulate several aspects of food intake. 

In humans, numerous clinical and epidemiological studies indicate that body weight and BMI are lower in cigarette smokers than in non-smokers [66,67,68,69,70,71]. Body leanness is particularly associated with the duration, rather than the intensity of smoking [66]. In addition, smoking cessation is usually accompanied by hyperphagia and weight gain which is more prominent in women [72,73,74,75]. Nicotine administration to both smokers and non-smokers does not change hunger sensations [64]. Despite this, it decreases meal size, without substantial changes in meal number [76,77]. However, given the health and addiction issues surrounding smoking, it is unlikely that non-prescription nicotine based weight control products could enter the market without considerable demonstration of efficacy and absence of psychological side effects both during treatment and at cessation. At the moment, nicotine preparations are almost exclusively used to delay post cessation weight gain [78,79,80]. 

### 4.3. Khat

The appetite suppressant effects of chewing leaves of the khat plant (*Catha edulis*) have been reported for several centuries. [81,82,83]. The predominant active ingredients of *C. edulis* are cathinone (1-α-aminopropiophenone) and cathine (D-*nor*-pseudoephedrine). These compounds share similarities with amphetamine, with up to 90% being absorbed during chewing, predominantly *via* the oral mucosa [84]. Amphetamine-like compounds affect appetite centrally, by acting in the hypothalamus. Apart from its central effect, cathinone enhances sympathomimetic activity leading to a delay in gastric emptying [85]. In healthy volunteers, khat chewing decreased hunger and increased fullness scores, this was associated to a prolonged gastric emptying which was significant when compared to lettuce chewing [86].

### 4.4. Hoodia Gordonii

*H. gordonii* (commercially available as pills, patches, and liquid: Hoodia pure ^®^ , Hoodia MAX ^®^ , Pure Hoodia^ ®^ , RapidSlim SX^ ®^, Hooderma^ ®^, Hoodia-HG57^ ®^), a member of the large milkweed family, is a desert-originating, succulent, slow growing plant [87]. Currently, there are more than twenty international patent applications/registrations on *H. gordonii* relating to the appetite suppressant, anti-diabetic activity and the treatment of gastric acid secretion [88]. The available literature offers limited reports on the biological effects of *H. gordonii* and its active compounds.

P57, the commercial extract of *H. gordonii* has been in the market for some time. It has been reported to increase ATP content in hypothalamic cells [89], which correlates with a decrease in appetite. This finding suggests that one mechanism of action of P57 is through a central mechanism; however an additional peripheral mechanism cannot be ruled out [90]. Phytopharm, who in 1997 were granted the license for the patent for the active component of the Hoodia P57 extract, have recently disclosed phase 1 studies in healthy overweight humans where significant reductions in caloric intake and body weight were achieved [91]. 

### 4.5. Caralluma Fimbriata

*C. fimbriata* (Slimaluma^®^) is an edible succulent cactus that belongs to the family Asclepiadaceae. Its key ingredients are pregnane glycosides, flavone glycosides, megastigmane glycosides, bitter principles, saponins and various other flavonoids [92]. The appetite suppressant action of *C. fimbriata* could be mainly attributed to the pregnane glycosides. These compounds seem to have peripheral and central effects. In the adipose tissue, pregnane glycosides reduce lipogenesis [93,94]. In the central structures regulating appetite, pregnane glycosides and its related molecules seem to share a similar mechanism of that of *H. gordonii* where they act by amplifying the signalling of the energy sensing function in the hypothalamus [89,95].

In overweight humans, two months administration of *C. fimbriata* extracts lead to a reduction in self-reported measures of appetite, body weight and waist circumference when compared to a control group [95]. Interestingly *C. fimbriata* selectively reduced the intake of refined sugars, sweets, cholesterol and saturated fats, without altering fruit, vegetable or fish intake. This could provide an additional mechanism of reduction in body weight since the consumption of foods such as whole grains, fruit and vegetables has been found to be directly associated with reduction in hunger and increased satiety levels, which could lead to lowered voluntary EI [96].

### 4.6. Coleus Forskohlii

*C. forskohlii* (ForsLean^®^) is a plant rich in alkaloids that belongs to the mint family [97]. One of the main active compounds in *C*. *forskohlii* is forskolin, a diterpene that acts directly on adenylate cyclase [98]. Adenylate cyclase is an enzyme that activates cyclic adenosine monophosphate (cAMP). In turn cAMP promotes lipolysis, increases the body's basal metabolic rate, and increases utilisation of body fat [99]. Administration of *C*. *forskohlii* extract to overweight women mitigated weight gain with no significant side effects, however, weight loss was not achieved [100]. Since this preparation affected weight gain further clinical study of *C*. *forskohlii* may be worthwhile. 

## 5. Phytochemicals that Increase Appetite and Body Weight

### 5.1. Cannabis Sativa

Although the use of cannabis (*C. sativa*) for medicinal and other purposes dates back at least four thousand years, understanding of the underlying pharmacology dates back only forty years. Cannabinoids are known for their rewarding effects and for their ability to stimulate increases in food intake (e.g., the marijuana 'munchies') [101]. Cannabis hyperphagia is largely attributable to Δ^9^-THC actions at brain CB1 cannabinoid receptor. 

After acute and chronic administration (typically in the form of cannabis cigarettes, and less frequently oral Δ^9^-THC administration), healthy volunteers (often experienced marijuana users) increase the consumption of mainly snack food which leads to a substantial increase in caloric intake, [101,102,103,104,105,106,107]. However, it has recently been demonstrated that Δ^9^-THC can have broad, dose-related effects on appetite that are not restricted to specific flavours or food types (Townson and Kirkham, unpublished observations). Δ^9^-THC seems to predominantly enhance the hedonic value of food [108,109]. A small number of clinical trials have assessed the possible benefits of cannabinoids in the treatment of wasting and appetite loss in cancer cachexia and AIDS. Treatment with Δ^9^-THC improved appetite ratings, increased food intake, and attenuated weight loss or induced weight gain [110,111,112,113]. 

### 5.2. Sutherlandia (Lessertia frutescens)

Sutherlandia *(L. frutescens)* is commonly given in the belief that this herb will treat some of the symptoms associated with HIV/AIDS, such as nausea and lack of appetite, amongst others [114]. A recent randomized, double-blind, placebo-controlled trial of *Sutherlandia* leaf powder in healthy adults showed that 800 mg/d during three months increased appetite ratings [115]. The constraints of the investigation related to limited sample size, precluding firm conclusions from being drawn about these preliminary data or any speculation related to mechanisms of action.

## 6. Conclusions

It can be concluded that to date there is no single phytochemical that can be considered an effective weight control product. Some of the phytochemicals reviewed above show potentially promising effects for weight control. However, for the majority of compounds and extracts, more data are needed to define the actual magnitude of effects and optimal doses. In addition, even if being proven effective, the use of some compounds (e.g., caffeine, nicotine, Δ^9^-THC, *C. aurantium*) is precluded due to safety issues.

This review focused on phytochemicals that affect human appetite and body weight, however, it is important to note that a far more greater amount of these compounds are currently under investigation in preclinical settings (*in vitro* and/or animal models). Even though the products here described have made it to clinical trial or commercialization phases, for the majority of them, the data available relating to the mechanism of action and benefits for weight control is still inconsistent or incomplete. For some products is not yet even established which aspects of energy balance (intake, uptake, or expenditure) are actually affected. 

Additionally, it is important to note here that although some phytochemicals that can be acquired over the counter have been scientifically tested, others have shown no proven efficacy.

Improved understanding and evidence on each of the reviewed and other proposed weight control ingredients will guide further research, as well as the selection of ingredients and product formats that can deliver the most attractive and effective benefits to consumers. Ultimately, only randomised, double blinded, placebo-controlled clinical trials of phytochemicals in humans can demonstrate their true potential. With regard to appetite and food intake this will involve proving that phytochemical based products significantly reduce daily caloric intake by adjusting appetite rather than by causing behavioural disruption or inducing malaise. For these substances to be catalogued as useful weight control management, significant placebo subtracted weight loss needs be demonstrated at least in the medium term e.g., up to 24 weeks of use.

## References

[1] Alper C.M., Mattes R.D. (2000). Effects of chronic peanut consumption on energy balance and hedonics. Int. J. Obes. Relat. Metab. Disord..

[2] Pasman W.J., Heimerikx J., Rubingh C.M., Van den Berg R., O'Shea M., Gambelli L., Hendriks H.F., Einerhand A.W., Scott C., Keizer H.G., Mennen L.I. (2008). The effect of Korean pine nut oil on *in vitro* CCK release, on appetite sensations and on gut hormones in post-menopausal overweight women. Lipids Health Dis..

[3] McLaughlin J., Grazia Luca M., Jones M.N., D'Amato M., Dockray G., Thompson D.G. (1999). Fatty acid chain length determines cholecystokinin secretion and effect on human gastric motility. Gastroenterology.

[4] Lawton C.L., Delargy H.J., Brockman J., Smith F.C., Blundell J.E. (2000). The degree of saturation of fatty acids influences post-ingestive satiety. Br. J. Nutr..

[5] Scott C., Pasman W., Hiemerikx J., Rubingh C., Van Den Berg R., O'Shea M., Gambelli L., Hendricks H., Mennen L., Einerhand A. (2007). Pinnothin™ suppresses appetite in overweight women. Appetite.

[6] Hughes G.M., Boyland E.J., Williams N.J., Mennen L., Scott C., Kirkham T.C., Harrold J.A., Keizer H.G., Halford J.C. (2008). The effect of Korean pine nut oil (PinnoThin) on food intake, feeding behaviour and appetite: a double-blind placebo-controlled trial. Lipids Health Dis..

[7] Burns A.A., Livingstone M.B., Welch R.W., Dunne A., Rowland I.R. (2002). Dose-response effects of a novel fat emulsion (Olibra) on energy and macronutrient intakes up to 36 h post-consumption. Eur. J. Clin. Nutr..

[8] Diepvens K., Soenen S., Steijns J., Arnold M., Westerterp-Plantenga M. (2007). Long-term effects of consumption of a novel fat emulsion in relation to body-weight management. Int. J. Obes. (Lond)..

[9] Welch I., Saunders K., Read N.W. (1985). Effect of ileal and intravenous infusions of fat emulsions on feeding and satiety in human volunteers. Gastroenterology.

[10] Welch I.M., Sepple C.P., Read N.W. (1988). Comparisons of the effects on satiety and eating behaviour of infusion of lipid into the different regions of the small intestine. Gut.

[11] Diepvens K., Steijns J., Zuurendonk P., Westerterp-Plantenga M.S. (2008). Short-term effects of a novel fat emulsion on appetite and food intake. Physiol. Behav..

[12] Burns A.A., Livingstone M.B., Welch R.W., Dunne A., Robson P.J., Lindmark L., Reid C A., Mullaney U., Rowland I.R. (2000). Short-term effects of yoghurt containing a novel fat emulsion on energy and macronutrient intakes in non-obese subjects. Int. J. Obes. Relat. Metab. Disord..

[13] Lewis Y.S., Neelakantan S. (1965). (−)-Hydroxycitric acid - the principal acid in the fruits of Garcinia cambogia. Desr. Psytochem..

[14] Clouatre D., Rosenbaum M.E. (1994). The Diet and Health Benefits of HCA.

[15] Sergio W. (1988). A natural food, the Malabar Tamarind, may be effective in the treatment of obesity. Med. Hypotheses.

[16] McCarty M., Majeed M., Majeed M., Rosen R., McCarty M., Conte A., Patil D., Butrym E. (1994). The pharmacology of Citrin. Citrin^®.^ A Revolutionary, Herbal Approach to Weight Management.

[17] Mattes R.D., Bormann L. (2000). Effects of (-)-hydroxycitric acid on appetitive variables. Physiol. Behav..

[18] Heymsfield S.B., Allison D., Vasselli J.R., Pietrobelli A., Greenfield D., Nuñez C. (1998). Garcinia cambogia (hydroxycitric acid) as a potential antiobesity agent: a randomized controlled trial. JAMA.

[19] Westerterp-Plantenga M.S., Kovacs E.M. (2002). The effect of (-)-hydroxycitrate on energy intake and satiety in overweight humans. Int. J. Obes. Relat. Metab. Disord..

[20] Preuss H.G., Rao C.V., Garis R., Bramble J.D., Ohia S.E., Bagchi M., Bagchi D. (2004). An overview of the safety and efficacy of a novel, natural(-)-hydroxycitric acid extract (HCA-SX) for weight management. J. Med..

[21] Greenwood M.R., Cleary M.P., Gruen R., Blase D., Stern J.S., Triscari J., Sullivan A.C. (1981). Effect of (-)-hydroxycitrate on development of obesity in the Zucker obese rat. Am. J. Physiol..

[22] Korenkov M., Sauerland S., Junginger T. (2005). Surgery for obesity. Curr. Opin. Gastroenterol..

[23] Han L.K., Kimura Y., Kawashima M., Takaku T., Taniyama T., Hayashi T., Zheng Y.N., Okuda H. (2001). Anti-obesity effects in rodents of dietary teasaponin, a lipase inhibitor. Int. J. Obes..

[24] Muramatsu K., Fukuyo M., Hara Y. (1986). Effect of green tea catechins on plasma cholesterol level in cholesterol-fed rats. J. Nutr. Sci. Vitaminol..

[25] Nagao T., Komine Y., Soga S., Meguro S., Hase T., Tanaka Y., Tokimitsu I. (2005). Ingestion of a tea rich in catechins leads to a reduction in body fat and malondialdehyde-modified LDL in men. Am. J. Clin. Nutr..

[26] Wolfram S., Wang Y., Thielecke F. (2006). Anti-obesity effects of green tea: From bedside to bench. Mol. Nutr. Food Res..

[27] Sano M., Tabata M., Suzuki M., Degawa M., Miyase T., Maeda-Yamamoto M. (2001). Simultaneous determination of twelve tea catechins by high-performance liquid chromatography with electrochemical detection. Analyst.

[28] Mandel S.A., Amit T., Weinreb O., Reznichenko L., Youdim M.B. (2008). Simultaneous manipulation of multiple brain targets by green tea catechins: A potential neuroprotective strategy for Alzheimer and Parkinson diseases. CNS Neurosci. Ther..

[29] Hase T., Komine Y., Meguro S., Takeda Y., Takahashi H., Matusi Y. (2001). Anti-obesity effects of tea catechins in humans. J. Oleo. Sci..

[30] Tsuchida T., Itakura H., Nakamura H. (2002). Reduction of body fat in humans by long-term ingestion of catechins. Prog. Med..

[31] Kovacs E.M., Mela D.J. (2006). Metabolically active functional food ingredients for weight control. Obes. Rev..

[32] Auvichayapat P., Prapochanung M., Tunkamnerdthai O., Sripanidkulchai B.O., Auvichayapat N., Thinkhamrop B., Kunhasura S., Wongpratoom S., Sinawat S., Hongprapas P. (2008). Effectiveness of green tea on weight reduction in obese Thais: A randomized, controlled trial. Physiol. Behav..

[33] Nagao T., Hase T., Tokimitsu I. (2007). A green tea extract high in catechins reduces body fat and cardiovascular risks in humans. Obesity (Silver Spring).

[34] Maki K.C., Reeves M.S., Farmer M., Yasunaga K., Matsuo N., Katsuragi Y., Komikado M., Tokimitsu I., Wilder D., Jones F., Blumberg J.B., Cartwright Y. (2009). Green tea catechin consumption enhances exercise-induced abdominal fat loss in overweight and obese adults. J. Nutr..

[35] Diepvens K., Kovacs E.M., Vogels N., Westerterp-Plantenga M.S. (2006). Metabolic effects of green tea and of phases of weight loss. Physiol. Behav..

[36] Chantre P., Lairon D. (2002). Recent findings of green tea extract AR25 (Exolise) and its activity for the treatment of obesity. Phytomedicine.

[37] Dulloo A.G., Duret C., Rohrer D., Girardier L., Mensi N., Fathi M., Chantre P., Vandermander J. (1999). Efficacy of a green tea extract rich in catechin polyphenols and caffeine in increasing 24-h energy expenditure and fat oxidation in humans. Am. J. Clin. Nutr..

[38] Rumpler W., Seale J., Clevidence B., Judd J., Willey E., Yamamoto S., Komatsu T., Sawaki T., Ishikura Y., Hosoda K. (2001). Oolong tea increases metabolic rate and fat oxidation in men. J. Nutr..

[39] Rudelle S., Ferruzzi M.G., Cristiani I., Moulin J., Mace K., Acheson K.J., Tappy L.  (2007). Effect of a thermogenic beverage on 24-hour energy metabolism in humans. Obesity (Silver Spring).

[40] del Castillo M.D., Ames J.M., Gordon M.H. (2002). Effect of roasting on the antioxidant activity of coffee brews. J. Agric. Food Chem..

[41] Thom E. (2007). The effect of chlorogenic acid enriched coffee on glucose absorption in healthy volunteers and its effect on body mass when used long-term in overweight and obese people. J. Int. Med. Res..

[42] Pellati F., Benvenuti S., Melegari M., Firenzuoli F. (2002). Determination of adrenergic agonists from extracts and herbal products of Citrus aurantium L. var. amara by LC. J. Pharm. Biomed. Anal..

[43] Preuss H.G., DiFerdinando D., Bagchi M., Bagchi D. (2002). Citrus aurantium as a thermogenic, weight-reduction replacement for ephedra: An overview. J. Med..

[44] Astrup A. (2000). Thermogenic drugs as a strategy for treatment of obesity. Endocrine.

[45] National Toxicology Program (1986). NTP toxicology and carcinogenesis studies of ephedrine sulfate (CAS, 134-72-5) in F344/N rats and B6C3F1 mice (Feed Studies). Natl. Toxicol. Program Tech. Rep. Series.

[46] Haaz S., Fontaine K.R., Cutter G., Limdi N., Perumean-Chaney S., Allison D.B. (2006). Citrus aurantium and synephrine alkaloids in the treatment of overweight and obesity: An update. Obes. Rev..

[47] Fredholm B.B., Bättig K., Holmén J., Nehlig A., Zvartau E.E. (1999). Actions of caffeine in the brain with special reference to factors that contribute to its widespread use. Pharmacol. Rev..

[48] Quarta D., Borycz J., Solinas M., Patkar K., Hockemeyer J., Ciruela F., Lluis C., Franco R., Woods A.S., Goldberg S.R., Ferré S. (2004). Adenosine receptor-mediated modulation of dopamine release in the nucleus accumbens depends on glutamate neurotransmission and N-methyl-D-aspartate receptor stimulation. J. Neurochem..

[49] Diepvens K., Westerterp K.R., Westerterp-Plantenga M.S. (2007). Obesity and thermogenesis related to the consumption of caffeine, ephedrine, capsaicin, and green tea. Am. J. Physiol. Regul. Integr. Comp. Physiol..

[50] Astrup A., Breum L., Toubro S., Hein P., Quaade F. (1992). The effect and safety of an ephedrine/caffeine compound compared to ephedrine, caffeine and placebo in obese subjects on an energy restricted diet. A double blind trial. Int. J. Obes. Relat. Metab. Disord..

[51] Pasman W.J., Westerterp-Plantenga M.S., Saris W.H. (1997). The effectiveness of long-term supplementation of carbohydrate, chromium, fibre and caffeine on weight maintenance. Int. J. Obes. Relat. Metab. Disord..

[52] Westerterp-Plantenga M.S., Lejeune M.P., Kovacs E.M. (2005). Body weight loss and weight maintenance in relation to habitual caffeine intake and green tea supplementation. Obes. Res..

[53] Tremblay A., Masson E., Leduc S., Houde A., Despres J.P. (1988). Caffeine reduces spontaneous energy intake in men but not in women. Nutr. Res..

[54] Racotta S., Leblanc J., Richard D. (1994). The effect of caffeine on food intake in rats: Involvement of corticotropin-releasing factor and the sympatho-adrenal system. Pharmacol. Biochem. Behav..

[55] Comer S.D., Haney M., Foltin R.W., Fischman M.W. (1997). Effects of caffeine withdrawal on humans living in a residential laboratory. Exp. Clin. Psychopharmacol..

[56] Belza A., Toubro S., Astrup A. (2009). The effect of caffeine, green tea and tyrosine on thermogenesis and energy intake. Eur. J. Clin. Nutr..

[57] Jessen A., Buemann B., Toubro S., Skovgaard I.M., Astrup A. (2005). The appetite-suppressant effect of nicotine is enhanced by caffeine. Diab. Ob. Metab..

[58] Lopez-Garcia E., van Dam R.M., Rajpathak S., Willett W.C., Manson J.E., Hu F.B. (2006). Changes in caffeine intake and long-term weight change in men and women. Am. J. Clin. Nutr..

[59] Acheson K.J., Zahorska-Markiewicz B., Pittet P., Anantharaman K., Jequier E. (1980). Caffeine and coffee: Their influence on metabolic rate and substrate utilization in normal weight and obese individuals. Am. J. Clin. Nutr..

[60] Jung R.T., Shetty P.S., James W.P., Barrand M.A., Callingham B.A. (1981). Caffeine: Its effect on catecholamines and metabolism in lean and obese humans. Clin. Sci. (Lond.).

[61] Hollands M.A., Arch J.R., Cawthorne M.A. (1981). A simple apparatus for comparative measurements of energy expenditure in human subjects: The thermic effect of caffeine. Am. J. Clin. Nutr..

[62] Graham T.E. (2001). Caffeine and exercise: Metabolism, endurance and performance. Sports Med..

[63] Taylor P., Oillman A.G., Goodman L., Rail T.W., Murad F. Ganglionic stimulating and blocking agents. The Pharmacological Basis of Therapeutics.

[64] Perkins K.A., Epstein L.H, Stiller R.L., Fernstrom M.H., Sexton J.E., Jacob R.G., Solberg R. (1991). Acute effects of nicotine on hunger and caloric intake in smokers and nonsmokers. Psychopharmacology.

[65] Dallosso H.M., James W.P. (1984). The role of smoking in the regulation of energy balance. Int. J. Obes..

[66] Albanes D., Jones D.Y., Micozzi M.S., Mattson M.E. (1987). Associations between smoking and body weight in the U.S. population: Analysis of NHANES II. Am. J. Public Health.

[67] Jo Y.H., Talmage D.A., Role L.W. (2002). Nicotinic receptor-mediated effects on appetite and food intake. J. Neurobiol..

[68] Williamson D.F., Madans J., Anda R.F., Kleinman J.C., Giovino G.A., Byers T. (1991). Smoking cessation and severity of weight gain in a national cohort. N. Engl. J. Med..

[69] Shimokata H., Muller D.C., Andres R. (1989). Studies in the distribution of body fat. III. Effects of cigarette smoking. JAMA.

[70] Flegal K.M., Troiano R.P., Pamuk E.R., Kuczmarski R.J., Campbell S.M. (1995). The influence of smoking cessation on the prevalence of overweight in the United States. N. Engl. J. Med..

[71] Huot I., Paradis G., Ledoux M. (2004). Quebec Heart Health Demonstration Project Research Group. Factors associated with overweight and obesity in Quebec adults. Int. J. Obes. Relat. Metab.Disord..

[72] Grunberg N.E., Bowen D.J., Winders S.E. (1986). Effects of nicotine on body weight and food consumption in female rats. Psychopharmacology (Berl.).

[73] Klesges R.C., Meyers A.W., Klesges L.M., La Vasque M.E. (1989). Smoking, body weight, and their effects on smoking behavior: A comprehensive review of the literature. Psychol. Bull..

[74] Pomerleau C.S., Seidman D.F., Covey L.S. (1999). Issues for women who wish to stop smoking. Helping the hard-core smoker.

[75] Pomerleau C.S., Pomerleau O.F., Namenek R.J., Mehringer A.M. (2000). Short-term weight gain in abstaining women smokers. J. Subst. Abuse Treat..

[76] Blaha V., Yang Z.J., Meguid M., Chai J.K., Zadak Z. (1998). Systemic nicotine administration suppresses food intake *via* reduced meal sizes in both male and female rats. Acta Med..

[77] Miyata G., Meguid M.M., Varma M., Fetissov S.O., Kim H.J. (2001). Nicotine alters the usual reciprocity between meal size and meal number in female rat. Physiol. Behav..

[78] Filozof C., Fernandez Pinilla M.C., Fernandez-Cruz A. (2004). Smoking cessation and weight gain. Obes. Rev..

[79] Gross J., Stitzer M.L., Maldonado J. (1989). Nicotine replacement: Effects of postcessation weight gain. J. Consult. Clin. Psychol..

[80] Allen S.S., Hatsukami D., Brintnell D.M., Bade T. (2005). Effect of nicotine replacement therapy on post-cessation weight gain and nutrient intake: A randomized controlled trial of postmenopausal female smokers. Addict. Behav..

[81] Le Bras M., Fretillere Y. (1965). Les aspects mrdicaux de la consommation habituelle du Cath. Mdd. trop..

[82] Halbach H. (1972). Medical aspects of the chewing of khat leaves. Bull. World Health Org..

[83] Zelger J.L., Carlini E.A. (1980). Anorexigenic effects of two amines obtained from Catha edulis Forsk. (Khat) in rats. Pharmacol. Biochem. Behav..

[84] Toennes S.W., Harder S., Schramm M., Niess C., Kauert G.F. (2003). Pharmacokinetics of cathinone, cathine and norephedrine after the chewing of khat leaves. Br. J. Clin. Pharmacol..

[85] Heymann T.D., Bhupulan A., Zureikat N.E., Bomanji J., Drinkwater C., Giles P., Murray-Lyon I.M. (1995). Khat chewing delays gastric emptying of a semi-solid meal. Aliment. Pharmacol. Ther..

[86] Murray C.D., Le Roux C.W., Emmanuel A.V., Halket J.M., Przyborowska A.M., Kamm M.A., Murray-Lyon I.M. (2008). The effect of Khat (Catha edulis) as an appetite suppressant is independent of ghrelin and PYY secretion. Appetite.

[87] Van Beek T.A., Verpoorte R., Svendsen A.B., Leeuwenberg A.J., Bisset N.G.  (1984). Tabernaemontana L. (Apocynaceae): A review of its taxonomy, phytochemistry, ethnobotany and pharmacology. J. Ethnopharmacol..

[88] van Heerden F.R. (2008). Hoodia gordonii: A natural appetite suppressant. J. Ethnopharmacol..

[89] MacLean D.B., Luo L.G. (2004). Increased ATP content/production in the hypothalamus may be a signal for energy-sensing of satiety: Studies of the anorectic mechanism of a plant steroidal glycoside. Brain Res..

[90] MacLean D.B. (1985). Abrogation of peripheral cholecystokinin-satiety in the capsaicin treated rat. Regul. Pept..

[91] Phytopharm plc (2008). Phytopharm Open Offer and Placing Prospectus 080228..

[92] Bader A., Braca A., De Tommasi N., Morelli I. (2003). Further constituents from Caralluma negevensis. Phytochemistry.

[93] Preuss H.G., Bagchi D., Bagchi M., Rao C.V., Dey D.K., Satyanarayana S. (2004). Effects of a natural extract of (-)-hydroxycitric acid (HCA-SX) and a combination of HCA-SX plus niacin-bound chromium and Gymnema sylvestre extract on weight loss. Diabetes Obes. Metab..

[94] Preuss H.G. (2004). Report on the Safety of Caralluma Fimbriata and its Extract.

[95] Kuriyan R., Raj T., Srinivas S.K., Vaz M., Rajendran R., Kurpad A.V. (2007). Effect of Caralluma fimbriata extract on appetite, food intake and anthropometry in adult Indian men and women. Appetite.

[96] Roberts S.B., Heyman M.B. (2000). Dietary composition and obesity: Do we need to look beyond dietary fat?. J. Nutr..

[97] Caprioli J., Sears M. (1983). Forskolin lowers intraocular pressure in rabbits, monkeys, and man. Lancet.

[98] Burns T.W., Langley P.E., Terry B.E., Bylund D.B., Forte L.R.J. (1987). Comparative effects of forskolin and isoproterenol on the cyclic AMP content of human adipocytes. Life Sci..

[99] Litosch I., Hudson T.H., Mills I., Li S.Y., Fain J.N. (1982). Forskolin as an activator of cyclic AMP accumulation and lipolysis in rat adipocytes. Mol. Pharmacol..

[100] Henderson S., Magu B., Rasmussen C., Lancaster S., Kerksick C., Smith P., Melton C., Cowan P., Greenwood M., Earnest C., Almada A., Milnor P., Magrans T., Bowden R., Ounpraseuth S., Thomas A., Kreider R.B. (2005). Effects of coleus forskohlii supplementation on body composition and hematological profiles in mildly overweight women. J. Int. Soc. Sports Nutr..

[101] Greenberg I., Kuehnle J., Mendelson J.H., Bernstein J.G. (1976). Effects of marihuana use on body weight and caloric intake in humans. Psychopharmacology (Berl.).

[102] Foltin R.W., Brady J.V., Fischman M.W. (1986). Behavioral analysis of marijuana effects on food intake in humans. Pharmacol. Biochem. Behav..

[103] Foltin R.W., Fischman M.W., Byrne M.F.  (1988). Effects of smoked marijuana on food intake and body weight of humans living in a residential laboratory. Appetite.

[104] Haney M., Rabkin J., Gunderson E., Foltin R.W. (2005). Dronabinol and marijuana in HIV+ marijuana smokers: Acute effects on caloric intake and mood. Psychopharmacology (Berl.).

[105] Hart C.L., Ward A.S., Haney M., Comer S.D., Foltin R.W., Fischman M.W.  (2002). Comparison of smoked marijuana and oral D9-tetrahydrocannbinol in humans. Psychopharmacology (Berl.).

[106] Haney M., Ward A.S., Comer S.D., Foltin R.W., Fischman M.W. (1999). Abstinence symptoms following oral THC administration to humans. Psychopharmacology (Berl.).

[107] Haney M., Gunderson E.W., Rabkin J., Hart C.L., Vosburg S.K., Comer S.D., Foltin R.W. (2007). Dronabinol and marijuana in HIV-positive marijuana smokers. Caloric intake, mood, and sleep. J. Acquir. Immune Defic. Syndr. Hum. Retrovirol..

[108] Abel E.L. (1971). Effects of marijuana on the solution of anagrams, memory and appetite. Nature.

[109] Hollister L.E. (1971). Hunger and appetite after single doses of marihuana, alcohol, and dextroamphetamine. Clin. Pharmacol. Ther..

[110] Regelson W., Butler J.R., Schultz J., Braude M., Szara S. (1976). Delta-9-tetrahydrocannabinol as an effective antidepressant and appetitestimulating agent in advanced cancer patients. The Pharmacology of Marijuana.

[111] Beal J.E., Olson R., Laubenstein L., Morales J.O., Bellman P., Yangco B., Lefkowitz L., Plasse T.F., Shepard K.V. (1995). Dronabinol as a treatment for anorexia associated with weight loss in patients with AIDS. J. Pain Symptom Manage..

[112] Plasse T.F., Gorter R.W., Krasnow S.H., Lane M., Shepard K.V., Wadleigh R.G. (1991). Recent clinical experience with dronabinol. Pharmacol. Biochem. Behav..

[113] Struwe M., Kaempfer S., Geiger C., Pavia A.T., Plasse T.F., Shepard K.V., Ries K., Evans T.G. (1993). Effect of dronabinol on nutritional status in HIV infection. Ann. Pharmacother..

[114] van Wyk B.E., Albrecht C. (2008). A review of the taxonomy, ethnobotany, chemistry and pharmacology of Sutherlandia frutescens (Fabaceae). J. Ethnopharmacol..

[115] Johnson Q., Syce J., Nell H., Rudeen K., Folk W.R. (2007). A randomized, double-blind, placebo-controlled trial of Lessertia frutescens in healthy adults. PLoS Clin. Trials.

